# Influence of TiO_2_ Nanoparticles on Growth and Phenolic Compounds Production in Photosynthetic Microorganisms

**DOI:** 10.1155/2014/961437

**Published:** 2014-12-28

**Authors:** Mattia Comotto, Alessandro Alberto Casazza, Bahar Aliakbarian, Valentina Caratto, Maurizio Ferretti, Patrizia Perego

**Affiliations:** ^1^Department of Civil, Chemical and Environmental Engineering (DICCA), University of Genoa, Via Opera Pia 15, 16145 Genoa, Italy; ^2^Research Center of Biologically Inspired Engineering in Vascular Medicine and Longevity (BELONG), Via Montallegro 1, 16145 Genoa, Italy; ^3^Department of Chemistry and Industrial Chemistry (DCCI), University of Genoa, Via Dodecaneso 31, 16146 Genoa, Italy; ^4^CNR-SPIN, Corso Perrone 24, 16156 Genoa, Italy

## Abstract

The influence of titanium dioxide nanoparticles (pure anatase and 15% N doped anatase) on the growth of *Chlorella vulgaris*, *Haematococcus pluvialis*, and *Arthrospira platensis* was investigated. Results showed that pure anatase can lead to a significant growth inhibition of *C. vulgaris* and *A. platensis* (17.0 and 74.1%, resp.), while for *H. pluvialis* the nanoparticles do not cause a significant inhibition. Since in these stress conditions photosynthetic microorganisms can produce antioxidant compounds in order to prevent cell damages, we evaluated the polyphenols content either inside the cells or released in the medium. Although results did not show a significant difference in *C. vulgaris*, the phenolic concentrations of two other microorganisms were statistically affected by the presence of titanium dioxide. In particular, 15% N doped anatase resulted in a higher production of extracellular antioxidant compounds, reaching the concentration of 65.2 and 68.0 mg g_DB_^−1^ for *H. pluvialis* and *A. platensis*, respectively.

## 1. Introduction

Over the last few years, nanotechnologies have been developed in several directions attracting major government and commercial investments and considerable academic interests. Nanoparticles (NPs) of metal oxide, especially titanium dioxide (TiO_2_), are widely used in different fields like cosmetics for sunscreens based on its UV absorbent property [1], self-cleaning surfaces and paints, solar cells [2, 3], and catalysis stand on the photocatalytic effect [4–7]. The latter property of NPs has inspired new promising technologies for water and air purification systems. Such a semiconductive effect is due to the specific electronic structure and depends mainly on the concentration, dimension of the particles (in particular surface/volume ratio), and crystal structure. For example, TiO_2_ anatase has a greater photocatalytic activity in comparison with rutile structure [8–11]. The photocatalytic effect is based on oxidation reactions which take place on the surface of particles and allow the production of high reactive hydroxyl and hydroperoxide radicals which cause the degradation of organic matters. As a consequence of oxidation processes, nanoparticles of TiO_2_ have been shown to exhibit an antimicrobial activity against viruses, bacteria, and fungi [12, 13]. In our previous research [14] we tested successfully the effect of two different structures of TiO_2_ nanoparticles (synthetized rutile and commercial anatase) on deactivation of* Escherichia coli*.

In this framework, we decided to expand our knowledge about the effect of TiO_2_ NPs on the other kinds of microorganisms, like photosynthetic ones. Some microalgal species can cause environmental issues due to their rapid growth and accumulation, as in the case of harmful algal bloom, which consists in a rapid increase of algal population in an aquatic system, with the ability to produce toxins which are dangerous for marine population, humans, and livestock [15, 16]. However, to the best of our knowledge, only a few studies have been performed investigating the effect of NPs on the reduction of harmful microalgae from aqueous cultures.

The biotoxicity of nickel oxide NPs on* Chlorella vulgaris* was evaluated by Gong et al. [17]. Results of this study showed that the NiO nanoparticles had severe impacts on the algae, with an EC_50_ value after 72 h of 32.28 mg_NiO_ L^−1^. Sadiq et al. [18] investigated the inhibitory effect of titanium NPs on two algal species. They observed that, after 72 h, 16.12 mg L^−1^ and 21.20 mg L^−1^ of commercial anatase TiO_2_ NPs were required to reduce 50% of algal growth for* Chlorella* sp. and* Scenedesmus* sp., respectively.

This study aimed at investigating the influence of two different kinds of titanium dioxide (anatase and 15% nitrogen doped anatase) NPs, previously synthetized in our laboratory [19], on the growth of three different kinds of microorganisms: two microalgal species (*Chlorella vulgaris* and* Haematococcus pluvialis* of* Trebouxiophyceae* and* Chlorophyceae* class, resp.) and a cyanobacterium (*Arthospira platensis *of* Cyanophyceae* class). Since the photocatalytic effect of NPs is based on oxidation reactions, we assumed that phenolic compounds, as secondary metabolites, will be released from the cells as a consequence of stress condition. To confirm our hypothesis we quantified phenolic compounds, either released in the medium or inside the cells, by spectrophotometric method. Phenolic compounds with their antioxidant properties have gained considerable interests to be used in several nutraceutical products, like cosmetics, food, pharmacology, and herbal medicine [20–22].

## 2. Materials and Methods

### 2.1. Materials

In this study, two different kinds of titanium dioxide (anatase and 15% nitrogen (N) doped anatase) were used. Both forms were obtained by sol-gel synthesis [19]. Anatase was obtained from an aqueous solution of titanium isopropoxide and 2-propanol, which was stirred at room temperature for 4 hours. This homogeneous gel was subsequently dried for 12 hours at 100°C. Nitrogen doped anatase was synthesized by adding titanium isopropoxide and 2-propanol to an aqueous solution of NH_3_ (15%). The mixture was stirred for 4 hours at room temperature and then was dried at 100°C for 12 hours. Both powders were ground and calcined in a mitten at 350°C for 1 hour to complete the crystallization.

Two microalgal species, specifically* Chlorella vulgaris* CCAP 211 (Culture Collection of Algae and Protozoa, Argyll, UK) and* Haematococcus pluvialis* CCAP 34/1F (Culture Collection of Algae and Protozoa, Argyll, UK), and the cyanobacterium* Arthrospira* (*Spirulina*)* platensis* UTEX1926 (University of Texas Culture Collection, Austin, TX, USA) were used.* C. vulgaris* was grown in the Bold's Basal Medium [23] and* H. pluvialis* in the modified Bold's Basal Medium [24], while* A. platensis* was cultivated in Schlösser Medium [25].

All reagents for TiO_2_ synthesis (titanium isopropoxide and 2-propanol) and for colorimetric analysis (sodium carbonate, Folin-Ciocalteu reagent, and methanol) and salts for media preparation were purchased from Sigma-Aldrich (Sigma-Aldrich, Milan, Italy).

### 2.2. Characterization of TiO_2_ Nanoparticles

The crystal structure of TiO_2_ and 15% N doped TiO_2_ was confirmed by X-Ray Powder Diffraction (XRPD) technique using a Philips PW1830 diffractometer, Eindhoven, The Netherlands (Bragg-Brentano geometry; Cu K*α*; Ni filtered; range 20–80_ 2 h; step 0.025_ 2 h; sampling time 10 s); the crystalline structures were refined according to the Rietveld method using the Fullprof program. Transmission electron microscopy (TEM) was performed using a JEOL JEM 2010 (200 kV, Lanthanum Boride crystal) microscope (Tokyo, Japan).

### 2.3. Growth Experiment

TiO_2_ (anatase and 15% N doped anatase) was weighed in 250 mL-Erlenmeyer flasks with 150 mL of medium in order to have an initial concentration of 0.10 g L^−1^. The flasks with TiO_2_ and the respective media mentioned in Section 2.1 were sterilized in an autoclave for 20 min at 121°C in order to prevent any contamination during the early stages of growth. The growth was done using a refrigerated incubator series 6000 (Termaks, Milan, Italy) equipped with artificial light (70 *μ*E m^−2^ s^−1^) and the flasks were stirred with a shaker model KS250 basic (IKA, Cologne, Germany). All microorganisms were grown at 25°C and the initial concentration of biomass was about 0.1 grams of dry biomass per litre of medium (g_DB_ L_M_^−1^). Three samples were studied: control, sample with TiO_2_ anatase, and one with 15% N doped TiO_2_. Each test was carried out in triplicate. The cultivation was stopped when the concentration of biomass reached the stationary phase of growth (9, 15, and 21 days for* H. pluvialis*,* A. platensis*, and* C. vulgaris*, resp.).

Biomass was also characterized by optical microscope model DMSL equipped with DC 200 digital camera (LEIKA, Wetzlar, Germany) in order to evaluate possible cellular modifications due to titanium dioxide addition.

### 2.4. Biomass Concentration

Biomass concentration was determined daily by optical density using an UV-Vis spectrophotometer model Lambda 25 (Perkin Elmer, Milan, Italy). Every measurement was carried out in triplicate and the biomass concentration of* C. vulgaris*,* H. pluvialis*, and* A. platensis* was related to the optical density by the following equations (1), (2), and (3), respectively:

(1) $$\begin{array}{c} {\text{ABS}}_{625}=4.2030x\;\left(R^{2}=0.9900\right), \end{array}$$

(2) $$\begin{array}{c} {\text{ABS}}_{625}=4.5561x\;\left(R^{2}=0.9825\right), \end{array}$$

(3) $$\begin{array}{c} {\text{ABS}}_{560}=0.0024x+0.1129\;\left(R^{2}=0.9921\right), \end{array}$$

where ABS is the absorbance at specific wavelength and *x* is the biomass concentration in grams of dry biomass per litre of medium (g_DB_ L_M_^−1^). All the equations were obtained experimentally.

A solution of the respective medium with the same concentration of TiO_2_ of the samples was used as the blank sample. After growth, biomass was separated from medium by centrifugation (7500 rpm) and stored at −18°C for further analysis.

### 2.5. Extraction and Analysis of Phenolic Compounds

The phenolic compounds were quantified either inside the microorganism cells or released from the cells in the medium.

For phenolic compounds evaluation in cells, biomass was centrifuged at 7500 rpm for 10 min, using a centrifuge model 42426 (ALC, Milan, Italy) and then it was dried to a constant moisture of about 4-5%. Polyphenols were extracted from dry biomass with methanol (0.10 g dry biomass in 10 mL of methanol) for 30 min using an ultrasound bath (FALC UTA 90, Treviglio, Italy). Then the extraction was carried on in a closed vessel for 5 h at room temperature, under magnetic stirring. The suspension was centrifuged and total phenolic content was determined using a modified version of Folin-Ciocalteu method [26]. Phenolics amount was determined using (*R* ^2^ = 0.9940)

(4) $$\begin{array}{c} {\text{ABS}}_{725}=0.0017x, \end{array}$$

where ABS_725_ is the absorbance at a wavelength of 725 nm and *x* is the polyphenols concentration in micrograms of gallic acid equivalent on millilitre (*μ*g_GAE_ mL^−1^).

For the phenolic compounds evaluation in the medium, total phenolic (TP) content was determined using the same method described above with some modifications. Briefly, after centrifugation at 7500 rpm for 10 min, 1 mL of medium was added to 0.50 mL of Folin-Ciocalteu reagent; after mixing, 1 mL of saturated solution of Na_2_CO_3_ was added to the mixture and the sample was stored in the dark for 1 hour. TP content was calculated by (*R* ^2^ = 0.9967)

(5) $$\begin{array}{c} {\text{ABS}}_{725}=0.0047x+0.0908, \end{array}$$

where ABS_725_ is the absorbance at a wavelength of 725 nm and *x* is the polyphenols concentration in micrograms of gallic acid equivalent on millilitre (*μ*g_GAE_ mL^−1^).

For total polyphenol analysis, an UV-Vis spectrophotometer model Lambda 25 (Perkin Elmer, Milan, Italy) was used.

### 2.6. Kinetic of Growth

The specific growth rate was calculated by

(6) $$\begin{array}{c} \mu =\frac{1}{t}\ln\left(\frac{X_{m}}{X_{0}}\right), \end{array}$$

where *X*_*m*_ and *X*_0_ are the concentrations of biomass at the end and at the beginning of a batch run, respectively, and *t* is the duration of the run.

The polyphenols productivity was calculated by

(7) $$\begin{array}{c} \upsilon^{c}=\frac{\text{TP}\cdot X_{m}}{t}, \end{array}$$

where TP is the sum of total phenolic content in the medium and in the cells, *X*_*m*_ is the concentration of biomass at the end of the batch run, and *t* is the duration of the run.

### 2.7. Statistical Analysis

All the determinations were carried out in triplicate and the results were expressed as mean values and standard deviations. Influences of the various parameters were assessed by analysis of variance (ANOVA) and Tukey's post-hoc test. Multiple comparisons of the means were made by the least significant difference test at *P* ≤ 0.05. The Statistica v. 8.0 software (StatSoft, Tulsa, OK, USA) was used for the analysis.

## 3. Results and Discussion

### 3.1. Characterization of TiO_2_ Nanoparticles

**Figure 1:**
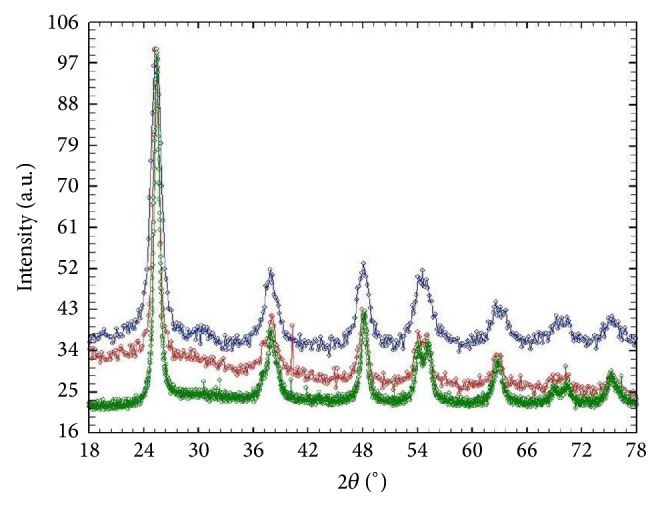
XRPD spectra of samples of anatase (red) and TiO_2_ 15% N doped anatase (blue) and anatase Pearson's crystal data (green).

The crystal structure of each TiO_2_ sample was identified by XRPD (Figure 1).

The XRPD patterns of TiO_2_ samples were matched with Pearson's crystal data, confirming that the titania nanoparticles had anatase phase predominantly.

**Figure 2:**
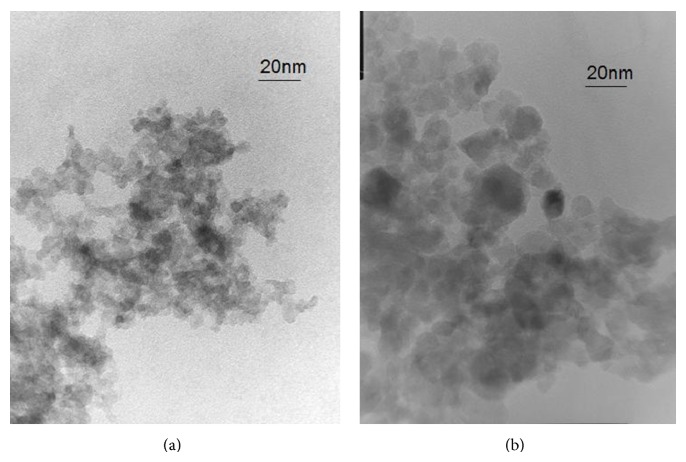
Transmission electron microscopic image of TiO_2_ anatase nanoparticles (a, 300x) and TiO_2_ 15% N doped anatase (b, 300x).

TEM analysis was carried out to confirm the primary size and shape of nanoparticles (Figure 2). The shape of both the pure anatase and 15% N doped anatase was nearly circular. This indicates that the process of nucleation and the phase of growth did not occur along a preferred direction. The grain size of pure anatase is 14.02 ± 2.18 nm, while for 15% N doped anatase it is 17.13 ± 2.00 nm; the particles have essentially the same size.

### 3.2. The Effect of TiO_2_ NPs on Growth of* C. vulgaris*,* H. pluvialis*, and* A. platensis*

**Figure 3:**
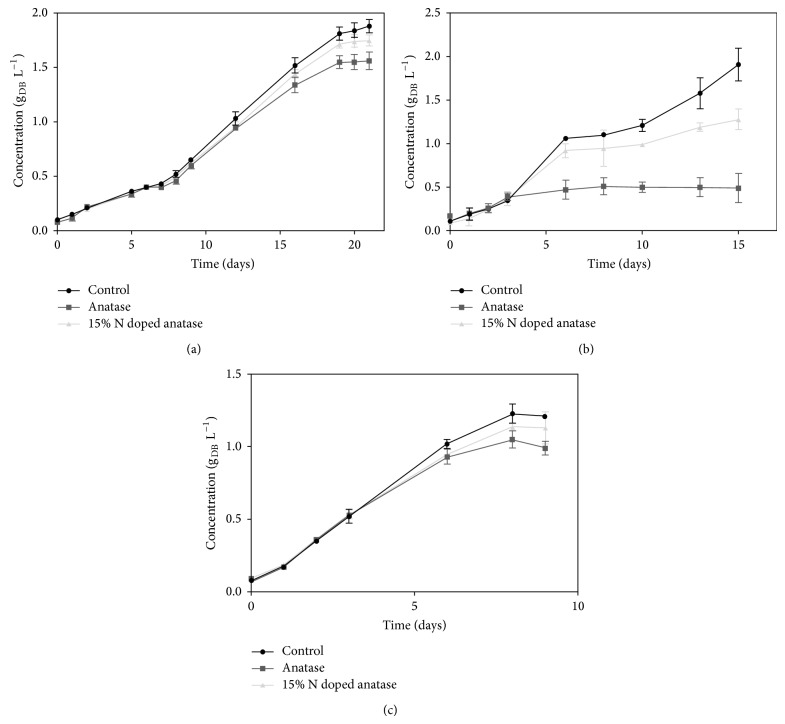
Effect of TiO_2_ NPs on the growth of* Chlorella vulgaris* (a),* Arthrospira platensis* (b), and* Haematococcus pluvialis* (c). (g_DB_ L^−1^ = grams of dried biomass per litre of media).

The effect of two types of TiO_2_ NPs addition on biomass growth is presented in Figure 3 and Table 1. Figure 3 shows the effect of TiO_2_ NPs on the growth of* C. vulgaris* (a),* H. pluvialis* (b), and* A. platensis* (c) till reaching the stationary phase, and Table 1 summarizes kinetic parameters.

Starting from the same initial concentration and after reaching the stationary phase of growth for each biomass, in the case of* C. vulgaris* 17.01% and 7.20% decreases in biomass concentration were noticed using pure anatase and 15% N doped anatase, respectively. The same effect was observed in the case of* H. pluvialis* (Figure 3(c)) in which the biomass concentration decreased 18.10% and 6.77% for anatase and 15% N doped anatase, respectively, while more significant (*P* < 0.05) inhibition effect of TiO_2_ NPs structure was seen for* A. platensis* compared to the aforementioned microalgal species. In this case, using 15% N doped anatase the inhibition was 32.94% and reached 74.09% with pure anatase. These results show that the effect of TiO_2_ NPs on the growth of photosynthetic microorganisms is related to the structure of the used nanoparticles since in all three microorganisms TiO_2_ NPs in the form of anatase inhibited the growth of biomass more than TiO_2_ 15% N doped form. We also noticed that the effect of TiO_2_ NPs on growth decrement was more significant (*P* < 0.05) only in the second phase of the exponential growth, while in the first phase it seemed to be negligible (these statistical differences were not shown in the figures). Significant statistical differences (*P* < 0.05) in the concentration of biomass for different treatments with respect to control tests were observed after 21 days and 13 days for* C. vulgaris* and* A. platensis*, respectively, while the use of NPs did not cause significant (*P* < 0.05) decrease for* H. pluvialis* at the end of the growth phase. In a similar study, Sadiq et al. [18] observed the inhibitory effect of nanotitania on* Chlorella* sp. growth. They confirmed that this inhibitory effect was dose-dependent to the titania NPs concentration. In their work, 192 mg L^−1^ of titania NPs resulted in more than 90% of algal cell population death after 72 hours.

**Figure 4:**
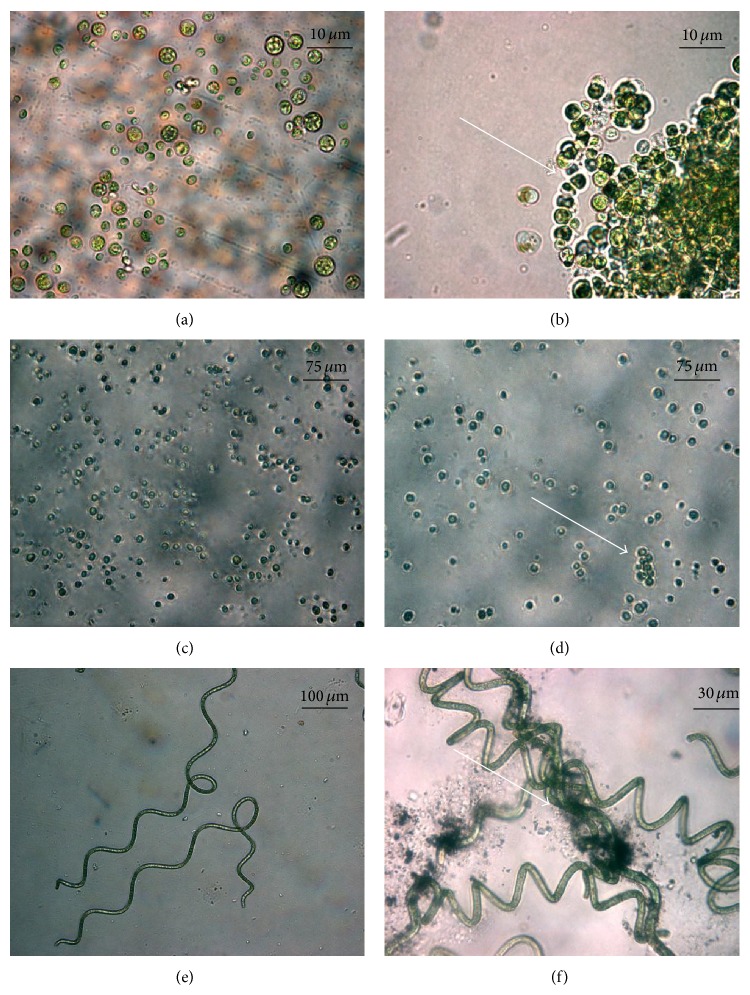
The effect of TiO_2_ NPs on cell morphology showed by phase contrast microscopic images. (a) and (b) show images of* Chlorella vulgaris *(100x); (c) and (d) show images of* Haematococcus pluvialis *(40x); (e) and (f) show images of* Arthrospira platensis *(40x and 100x, resp.). Left columns (a, c, and e) show the images for control tests of each microorganism while right columns (b, d, and f) show images of TiO_2_ NPs treated microorganisms. Arrow marks indicate the cellular aggregations and cellular wall degradation.

Concerning the growth rate (Table 1), both forms of TiO_2_ did not affect* C. vulgaris* concentration when compared to the control test, while a significant difference was obtained (*P* < 0.05) in the case of* H. pluvialis* and* A. platensis* using pure anatase with respect to the growth rate obtained without treatment (control). This is due to the different nature of the cells:* A. platensis* is a cyanobacterium and the different cellular structure might imply different response behaviour to the photocatalytic effect. In order to confirm the effect of TiO_2_ NPs on the cell morphology we used optical microscope to observe the treated biomasses (Figure 4).

Generally, the presence of titanium dioxide leads to a stress condition during the growth. In the case of* C. vulgaris* the normal growth (Figure 4(a)) leads to single and isolated cells, but the presence of NPs (Figure 4(b)) causes the formation of cellular aggregates. This effect was not so evident in the case of* H. pluvialis* (Figure 4(d)). Also, radicals generated by NPs should attack cells, leading to the cellular wall degradation with the leak of the intracellular material, as clearly shown in the case of* A. platensis* (Figure 4(f)).

### 3.3. The Effect of TiO_2_ NPs on Intra- and Extracellular Polyphenol Contents

It is known that when cells are exposed to environmental stresses, the antioxidant enzymes can be produced in order to protect cells from free radical damages [27]. Phenolic compounds are a group of antioxidants which might be a possible inhibition factor of the oxidative processes [21, 28–30]. Based on the mentioned prospective, we determined the concentration of phenolic compounds both inside the cells and released in the medium after treatment with TiO_2_ NPs (Table 2).

For* C. vulgaris* the presence of TiO_2_ NPs did not influence (*P* < 0.05) the extracellular phenolic concentration (decrease of 7.0% and 8.5% for 15% N doped TiO_2_ and TiO_2_ anatase, resp.). Although the concentration of intracellular phenolic compounds was not statistically (*P* < 0.05) affected by addition of TiO_2_ NPs, both pure anatase and 15% N doped anatase caused a decrease of phenols concentration of 17.0% and 22.1%, respectively, compared to the control test. Hajimahmoodi et al. [31] have shown similar results about the intracellular concentration (3.69 mg g_DB_^−1^ for the extracted aqueous fraction); since their growth has been done in similar condition but not under stress, we can definitely say that the influence of the titanium dioxide on* C. vulgaris* is negligible. Regarding* H. pluvialis*, the treatment with TiO_2_ anatase resulted in a release of considerable amount of phenolic compounds in the medium (116.13 mg g_DB_^−1^) with a decrease of intracellular concentration of 12.0% in comparison with control test, while the treatment with 15% N doped anatase resulted in an increase of phenolic content in the cells and in a decrease of extracellular phenolic concentration. This could be due to the high photocatalytic activity of TiO_2_ NPs in the form of N doped anatase which induces a higher production of phenolic compounds as a response to stress condition. Once released in the medium, the produced phenolic compounds were degraded in the presence of free radicals, confirming the extracellular phenolic compounds drop. The higher concentration of phenolic compounds released into the medium from* H. pluvialis* respective to* C. vulgaris* protects cells from degradation, as confirmed by optical microscope observation (Figure 4). Moreover, in contrast to the literature [32], our batches show higher concentration of the intracellular phenolic compounds. This suggests that the titanium dioxide, especially in the form of 15% N doped anatase, could have a positive influence in the production of these chemicals.

Finally, for* A. platensis* we obtained different results compared to other microorganisms: both forms of TiO_2_ showed an increase of phenolic content in the medium (127.2% and 452.2% for pure anatase and 15% N doped anatase, respectively). On the contrary, for intracellular concentration of phenolic compounds we obtained a relevant decrease compared to control, with a higher reduction caused by 15% N doped anatase (74.5%) compared to pure anatase (23.5%). Concerning the phenolic compounds productivity (intra- and extracellular), we noticed that the addition of TiO_2_ in the form of 15% N doped anatase resulted in increased (*P* < 0.05) phenol productivity in the case of* H. pluvialis* and* A. platensis* when compared to control tests, while both forms of TiO_2_ did not show significant influences on phenolic compounds productivity of* C. vulgaris*.

## 4. Conclusion

In conclusion, pure and 15% N doped TiO_2_ anatase were not able to totally inhibit the growth of* C. vulgaris*,* H. pluvialis*, and* A. platensis*. Nanoparticles caused only an inhibition during the second phase of the growth, which was higher in presence of pure anatase. In these situations, titanium dioxide leads to a stress condition resulting in a modification of the phenolic compounds made by the photosynthetic organisms. In particular, the extracellular release of polyphenols represents a starting point for further investigations since these compounds, after purification processes, could represent a natural antioxidant source for pharmaceutical and cosmetic products.

## Figures and Tables

**Table 1:** Effect of different TiO_2_ NPs on growth and kinetic parameters of different microorganisms.

Sample	*C. vulgaris *		*H. pluvialis *		*A. platensis *	
	Growth inhibition	*μ*	Growth inhibition	*μ*	Growth inhibition	*μ*
	(%)	(days^−1^)	(%)	(days^−1^)	(%)	(days^−1^)
Control	—	0.14 ± 0.00^a^	—	0.30 ± 0.01^b^	—	0.19 ± 0.00^a^
TiO_2_ 15% N doped	7.20^a^	0.14 ± 0.00^a^	6.77^a^	0.28 ± 0.02^ab^	32.94^a^	0.17 ± 0.00^a^
TiO_2_ anatase	17.01^b^	0.14 ± 0.00^a^	18.10^a^	0.27 ± 0.01^a^	74.09^b^	0.07 ± 0.02^b^

Means (*n* = 3) ± standard deviation with different letters (a and b) in the same column are significantly different (*P* < 0.05).

*μ* = specific growth rate.

**Table 2:** Total polyphenols content both in the medium (extracellular) and inside the cells (intracellular) and polyphenols productivity (*υ* ^*c*^) for each kind of photosynthetic microorganism treated with TiO_2_ NPs.

Sample	*C. vulgaris *			*H. pluvialis *			*A. platensis *		
	Extracellular concentration	Intracellular concentration	*υ* ^*c*^ (mg L^−1^ days^−1^)	Extracellular concentration	Intracellular concentration	*υ* ^*c*^ (mg L^−1^ days^−1^)	Extracellular concentration	Intracellular concentration	*υ* ^*c*^ (mg L^−1^ days^−1^)
	(mg g_DB_^−1^)	(mg g_DB_^−1^)		(mg g_DB_^−1^)	(mg g_DB_^−1^)		(mg g_DB_^−1^)	(mg g_DB_^−1^)	
Control	12.41 ± 1.20^a^	4.76 ± 1.14^a^	10.14 ± 0.60^a^	94.97 ± 7.06^b^	2.50 ± 0.91^a^	13.02 ± 0.12^a^	12.31 ± 0.72^a^	5.10 ± 0.14^a^	2.26 ± 0.06^b^
TiO_2_ 15% N doped	11.54 ± 1.60^a^	3.71 ± 0.23^a^	8.54 ± 0.22^a^	65.22 ± 1.59^a^	6.60 ± 0.93^b^	9.01 ± 0.25^b^	67.98 ± 1.97^c^	1.30 ± 0.09^c^	1.04 ± 0.04^c^
TiO_2_ anatase	11.36 ± 0.65^a^	3.95 ± 0.34^a^	8.31 ± 0.22^a^	116.13 ± 3.76^c^	2.20 ± 0.24^a^	13.01 ± 0.52^a^	27.97 ± 5.19^b^	3.90 ± 0.82^b^	5.87 ± 0.06^a^

Means (*n* = 3) ± standard deviation with different letters (a–c) in the same column are significantly different (*P* < 0.05).

Mg g_DB_^−1^ = milligrams per gram of dried biomass.

## References

[1] Kaida T., Kobayashi K., Adachi M., Suzuki F. (2004). Optical characteristics of titanium oxide interference film and the film laminated with oxides and their applications for cosmetics. *Journal of Cosmetic Science*.

[2] Burke A., Ito S., Snaith H., Bach U., Kwiatkowski J., Grätzel M. (2008). The function of a TiO_2_ compact layer in dye-sensitized solar cells incorporating “planar” organic dyes. *Nano Letters*.

[3] Dohrmann J. K., Schaaf N.-S. (1992). Energy conversion by photoelectrolysis of water: determination of efficiency by in situ photocalorimetry. *The Journal of Physical Chemistry*.

[4] Caballero L., Whitehead K. A., Allen N. S., Verran J. (2009). Inactivation of *Escherichia coli* on immobilized TiO_2_ using fluorescent light. *Journal of Photochemistry and Photobiology A: Chemistry*.

[5] Esterkin C. R., Negro A. C., Alfano O. M., Cassano A. E. (2005). Air pollution remediation in a fixed bed photocatalytic reactor coated with TiO_2_. *AIChE Journal*.

[6] Choi H., Stathatos E., Dionysiou D. D. (2006). Sol-gel preparation of mesoporous photocatalytic TiO_2_ films and TiO_2_/Al_2_O_3_ composite membranes for environmental applications. *Applied Catalysis B: Environmental*.

[7] Keshmiri M., Mohseni M., Troczynski T. (2004). Development of novel TiO_2_ sol-gel-derived composite and its photocatalytic activities for trichloroethylene oxidation. *Applied Catalysis B: Environmental*.

[8] Jang H. D., Kim S.-K., Kim S.-J. (2001). Effect of particle size and phase composition of titanium dioxide nanoparticles on the photocatalytic properties. *Journal of Nanoparticle Research*.

[9] Bacsa R. R., Kiwi J. (1998). Effect of rutile phase on the photocatalytic properties of nanocrystalline titania during the degradation of *p*-coumaric acid. *Applied Catalysis B: Environmental*.

[10] Chhabra V., Pillai V., Mishra B. K., Morrone A., Shah D. O. (1995). Synthesis, charaterization, and properties of microemulsion-mediated nanophase TiO_2_ particles. *Langmuir*.

[11] Ji J., Long Z., Lin D. (2011). Toxicity of oxide nanoparticles to the green algae *Chlorella sp.*. *Chemical Engineering Journal*.

[12] Matsunaga T., Tomoda R., Nakajima T., Wake H. (1985). Photoelectrochemical sterilization of microbial cells by semiconductor powders. *FEMS Microbiology Letters*.

[13] Blake D. M., Maness P. C., Huang Z., Wolfrum E. J., Huang J., Jacoby W. A. (1999). Application of the photocatalytic chemistry of titanium dioxide to disinfection and the killing of cancer cells. *Separation and Purification Methods*.

[14] Caratto V., Aliakbarian B., Casazza A. A., Setti L., Bernini C., Perego P., Ferretti M. (2013). Inactivation of *Escherichia coli* on anatase and rutile nanoparticles using UV and fluorescent light. *Materials Research Bulletin*.

[15] Pierce R. H., Henry M. S., Higham C. J., Blum P., Sengco M. R., Anderson D. M. (2004). Removal of harmful algal cells (*Karenia brevis*) and toxins from seawater culture by clay flocculation. *Harmful Algae*.

[16] Sengco M. R., Anderson D. M. (2004). Controlling harmful algal blooms through clay flocculation. *Journal of Eukaryotic Microbiology*.

[17] Gong N., Shao K., Feng W., Lin Z., Liang C., Sun Y. (2011). Biotoxicity of nickel oxide nanoparticles and bio-remediation by microalgae *Chlorella vulgaris*. *Chemosphere*.

[18] Sadiq I. M., Dalai S., Chandrasekaran N., Mukherjee A. (2011). Ecotoxicity study of titania (TiO_2_) NPs on two microalgae species: *Scenedesmus sp.* and *Chlorella sp.*. *Ecotoxicology and Environmental Safety*.

[19] Caratto V., Setti L., Campodonico S., Carnasciali M. M., Botter R., Ferretti M. (2012). Synthesis and characterization of nitrogen-doped TiO_2_ nanoparticles prepared by sol-gel method. *Journal of Sol-Gel Science and Technology*.

[20] Spolaore P., Joannis-Cassan C., Duran E., Isambert A. (2006). Commercial applications of microalgae. *Journal of Bioscience and Bioengineering*.

[21] Cardozo K. H. M., Guaratini T., Barros M. P. (2007). Metabolites from algae with economical impact. *Comparative Biochemistry and Physiology C: Toxicology and Pharmacology*.

[22] Li Y., Horsman M., Wu N., Lan C. Q., Dubois-Calero N. (2008). Biofuels from microalgae. *Biotechnology Progress*.

[23] Guillard R. R., Ryther J. H. (1962). Studies of marine planktonic diatoms. I. *Cyclotella nana* Hustedt, and *Detonula confervacea* (cleve) Gran. *Canadian Journal of Microbiology*.

[24] http://www.ccap.ac.uk/media/documents/3N_BBM_V_000.pdf.

[25] Schlösser U. G. (1982). Sammlung von Algenkulturen. *Bericht der Deutschen botanischen Gesellschaft*.

[26] Casazza A. A., Aliakbarian B., de Faveri D., Fiori L., Perego P. (2012). Antioxidants from winemaking wastes: a study on extraction parameters using response surface methodology. *Journal of Food Biochemistry*.

[27] Li M., Hu C., Zhu Q., Chen L., Kong Z., Liu Z. (2006). Copper and zinc induction of lipid peroxidation and effects on antioxidant enzyme activities in the microalga Pavlova viridis (Prymnesiophyceae). *Chemosphere*.

[28] Aliakbarian B., Dehghani F., Perego P. (2009). The effect of citric acid on the phenolic contents of olive oil. *Food Chemistry*.

[29] Ben Hamissa A. M., Seffen M., Aliakbarian B., Casazza A. A., Perego P., Converti A. (2012). Phenolics extraction from *Agave americana* (L.) leaves using high-temperature, high-pressure reactor. *Food and Bioproducts Processing*.

[30] Palmieri D., Aliakbarian B., Casazza A. A., Ferrari N., Spinella G., Pane B., Cafueri G., Perego P., Palombo D. (2012). Effects of polyphenol extract from olive pomace on anoxia-induced endothelial dysfunction. *Microvascular Research*.

[31] Hajimahmoodi M., Faramarzi M. A., Mohammadi N., Soltani N., Oveisi M. R., Nafissi-Varcheh N. (2010). Evaluation of antioxidant properties and total phenolic contents of some strains of microalgae. *Journal of Applied Phycology*.

[32] Goiris K., Muylaert K., Fraeye I., Foubert I., de Brabanter J., de Cooman L. (2012). Antioxidant potential of microalgae in relation to their phenolic and carotenoid content. *Journal of Applied Phycology*.

